# Early echocardiographic assessment of cardiac function may be prognostically informative in unresuscitated patients with sepsis: A prospective observational study

**DOI:** 10.1371/journal.pone.0269814

**Published:** 2022-07-08

**Authors:** Robert R. Ehrman, Mark J. Favot, Nicholas E. Harrison, Lyudmila Khait, Jakob E. Ottenhoff, Robert D. Welch, Phillip D. Levy, Robert L. Sherwin

**Affiliations:** 1 Department of Emergency Medicine, Wayne State University School of Medicine, Detroit, Michigan, United States of America; 2 Department of Emergency Medicine, Integrative Biosciences Center, Wayne State University School of Medicine, Detroit, Michigan, United States of America; Azienda Ospedaliero Universitaria Careggi, ITALY

## Abstract

**Purpose:**

The goal of this study was to explore the association cardiac function at Emergency Department (ED) presentation prior to the initiation of resuscitation, and its change at 3-hours, with adverse outcomes in patients with sepsis.

**Methods:**

This was a prospective observational study of patients presenting to an urban ED with suspected sepsis. Patients had a point-of-care echocardiogram performed prior to initiation of resuscitation and again 3 hours later. Left-ventricular (LV) parameters recorded included e’, and E/e’, and ejection fraction (EF); right-ventricular (RV) function was evaluated using tricuspid annular plane systolic excursion (TAPSE). Logistic and generalized linear regression were used to assess the association of echocardiographic parameters and ≥ 2-point increase in SOFA score at 24 hours (primary outcome) and 24-hours SOFA score and in-hospital mortality (secondary outcomes).

**Results:**

For ΔSOFA ≥ 2 and 24-hour SOFA score, declining LVEF was associated with better outcomes in patients with greater baseline SOFA scores, but worse outcomes in patients with lower baseline scores. A similar relationship was found for ΔTAPSE at 3 hours. Reduced LVEF at presentation was associated with increased mortality after adjusting for ED SOFA score (odds-ratio (OR) 0.76 (CI 0.60–0.96). No relationship between diastolic parameters and outcomes was found. IVF administration was similar across ΔLVEF/TAPSE sub-groups.

**Conclusions:**

Our results suggest that early change in LV and RV systolic function are independently prognostic of sepsis illness severity at 24-hours. Further study is needed to determine if this information can be used to guide treatment and improve outcomes.

## Introduction

Cardiac dysfunction in sepsis was recognized more than four decades ago [1, 2], but the underlying pathophysiology and prognostic implications remain unclear. Lack of uniformity in design and reporting in existing studies has produced contradictory results despite the fact that a link between adverse outcomes and a litany of echocardiographic parameters have been investigated [3]. Delineation of the natural history of septic cardiomyopathy (SC) is a critical step towards creation of a formal definition of the disease and the long-term goal of development of targeted therapeutics.

The first description of SC was reduced left-ventricular ejection fraction (LVEF) that recovered after the septic insult [2]. Subsequent studies, however, failed to detect a meaningful relationship between LVEF and outcomes [4, 5]. Diastolic dysfunction—both E/e’ and e’ in isolation—has also been extensively investigated. Some studies report increased mortality as diastolic function worsens [6–10], while other report no such relationship [11–15].

A contributor to conflicting outcomes amongst existing studies is that nearly all enrolled patients in the Intensive Care Unit (ICU), with echocardiograms performed up to 48 hours after admission [3]. This leads to heterogeneity of observed echocardiography parameters as disease progression and antecedent treatments administered according to individual patient needs can alter cardiac function prior to initial echocardiograms. Capturing patients in the Emergency Department (ED), prior to initiation of therapeutic interventions, could provide additional clinically informative data.

To address these gaps in existing knowledge, we performed a prospective observational study of ED patients with sepsis, with initial echocardiograms performed at the time of initiation of resuscitative interventions. The primary aim of the study was to test the hypothesis that impaired diastolic mitral annular velocity (e’) at presentation would be associated with increased illness severity at 24-hours in patients with sepsis. The association between early change in e’ velocity, as well as left and right ventricular systolic function, and increased 24-hour illness severity, were assessed as secondary aims.

## Methods

This prospective observational study was performed at an urban Level 2 trauma center with approximately 100,000 ED visits per year and a three-year Emergency Medicine Residency program. The study was approved by the Wayne State University Institutional Review Board and performed in accordance with the standards of the Declaration of Helsinki. Manuscript preparation was in accordance with the Strengthening the Reporting of Observational Studies in Epidemiology (STROBE) guidelines [16].

### Selection of participants

Patients 18 years of age or older were eligible if they met the following inclusion criteria: ED clinician suspicion for infection requiring treatment, and either serum lactate >2mmol/L or systolic blood pressure ≤ 90mm/Hg. Exclusion criteria were pregnancy, transfer to another institution, incarceration, atrial or ventricular dysrhythmia requiring rate or rhythm control, presence of a left-ventricular (LV) assist device, or receipt of >1L IVF prior to performance of the initial study echocardiogram. Patients were enrolled as a convenience sample when a study sonographer was available.

### Image acquisition and interpretation

Study echocardiograms were performed by one of 4 study sonographers: 2 cardiac sonographers and 2 physicians, both of whom completed Emergency Ultrasound fellowship and have extensive experience with clinical and research echocardiography. All examinations were performed with a GE Vivid q ultrasound system (GE Healthcare, Milwaukee, WI) using a phased array transducer and continuous ECG monitoring. Each participant had two bedside echocardiograms (BE) performed: the first (0HrBE) at the time of enrollment, the second 3-hours later (3HrBE), to coincide with the Surviving Sepsis Campaign 3-hour bundle goal of administration of 30cc/kg of IVF [17]. Echocardiograms were interpreted offline by one of two study investigators (MJF, RRE) who were blinded to all clinical data at the time of interpretation. Both investigators are testamurs of the Examination of Special Competence in Adult Echocardiography administered by the National Board of Echocardiography.

Image acquisition was carried out in accordance with ASE guidelines for chamber quantification and assessment of diastolic function [18, 19]. For interpretation, LV diastolic function was assessed by E velocity, e’ velocity, and E/e’; LV systolic function was assessed by visual estimation of LVEF in increments of 5%, and RV systolic function by tricuspid annular plane systolic excursion (TAPSE). Collapse of the inferior vena cava (IVC) with respiration was recorded as ≥50% or <50%. The full imaging protocol is provided in the S1 File. Inter-rater reliability for e’, LVEF, and TAPSE was assessed using interclass correlation coefficient (ICC) on 20 random echocardiograms interpreted by both investigators (e’, ICC = 0.89 (CI 0.74–0.95); LVEF, ICC = 0.79 (CI 0.48–0.92); TAPSE, ICC = 0.86 (CI 0.64–0.94)).

### Data collection and storage

Clinical data were abstracted from the electronic medical record by trained research assistants and entered into a REDCap (v 10.3.3, Vanderbilt University) database following previously described guidelines [20]. Data collected included demographics, medical history, vital signs, laboratory results, and therapeutic interventions; unless otherwise stated, all reported data represent initial ED values and interventions. SOFA score was calculated using initial ED data and again at 24 hours, using worst post-ED values. SOFA variables that were not obtained were entered as 0; at 24 hours, any variables that were not repeated from initial measurement were imputed using a last-observation-carried forward approach [21]. For patients without direct measurement, FiO_2_ was calculated using previously described methods [22, 23].

The primary outcome was increased illness severity at 24-hours (defined as a ≥2-point increase in Sequential Organ Failure Assessment (SOFA) score of from the ED value. This was chosen as a clinically meaningful endpoint as ΔSOFA score ≥ 2 (DS2) is associated with an OR of in-hospital death of 7.5 for patients with a low baseline SOFA, 2.7 for patients with a high baseline, and a pooled OR of 4.0 [24, 25]). Secondary outcomes included illness severity at 24-hours (defined as peak post-ED SOFA score within 24-hours of enrollment) and in-hospital mortality. Patients who died in ≤24-hours were considered to have met the primary outcome.

### Statistical analysis

Patient characteristics, treatments administered, and outcomes are reported as means (with standard deviation [SD]), medians (with interquartile range [IQR]), or proportions (with 95% confidence intervals [CI]), as appropriate. Echocardiographic parameters were analyzed as continuous variables, with changes from baseline to 3 hours calculated as (3HrBE)—(0HrBE).

For the primary outcome, the association between DS2 and e’ velocity at presentation was assessed using logistic regression (LR), with adjustment for ED SOFA score and IVC collapse (as a proxy for preload/volume status. LR was also used to test change in e’ velocity from 0–3 hours as a secondary predictor of DS2. We also tested the Interaction between e’ and ED SOFA score for these models. Exploratory analyses, using LVEF, TAPSE and ΔLVEF/ΔTAPSE from 0–3 hours as individual predictors of DS2, were similarly performed. For the exploratory outcome of in-hospital mortality, the individual association between e’ velocity, LVEF, and TAPSE, at presentation, adjusted for ED SOFA score and IVC collapse were assessed using LR. Calibration of LR models was assessed by visual inspection of calibration plots from PROC LOGISTIC.

Inclusion of covariates in the LR models was limited due to the low number of events (n = 17 for death and n = 17 for DS2). Therefore, generalized linear models (GLMs) were used to explore the impact of ΔLVEF and ΔTAPSE (in separate models) from 0–3 hours on the outcome of 24-hour illness severity, measured by peak 24-hour SOFA score. Covariates were selected “by meaning”, and included age, ED SOFA score, change in average E/e’ (a proxy of LV filling pressure) and IVC collapsibility (a proxy of preload). ED lactate, troponin-I, and b-type natriuretic peptide (BNP) were considered for addition as factors potentially associated with illness severity, but not already included in the SOFA score. Interactions between echocardiographic parameters and ED SOFA score were tested. Statistical analysis was performed using SAS software, version 9.4 (SAS Institute, Cary, NC); two-tailed significance level was set at 0.05. Assessment of model fit and tuning details are provided in the S1 File.

### Sample size calculation

For 80% power, the calculated sample size was 95 patients to detect an OR of the primary outcome (ΔSOFA ≥ at 24 hours) of 1.35 per 1cm/sec decrease in e’ velocity, using LR with baseline SOFA score, IVF volume in 24 hours, and e’ velocity at presentation as predictors. The study was closed at the start of the SARS-CoV2 pandemic, prior to reaching enrollment targets, owing to cessation of clinical research at our institution at that time.

## Results

From August, 2018 to March, 2020, a total of 107 patients had an initial echocardiogram performed, of which 73 were included in the final analysis. Reasons for exclusion included inability to obtain consent (n = 21), refusal (n = 6), transfer to another hospital (n = 3), uncertainty as to the amount of IVF administered prior to BE1 (n = 2) development of rapid atrial fibrillation in the ED (n = 1), and corrupted image files (n = 1).

All patients were African-American, 31 patients (42%) were female, median hospital LOS was 6.5 days (IQR 7 days), 14 (19%) had a history of heart failure, and 19 (26%) had a history of end-stage renal disease. Seventeen patients (23%) met the primary outcome and in-hospital mortality was 23% (n = 17). Nineteen patients (26%) required endotracheal intubation, 19 (26%) required vasopressor support, and 29 (39%) were admitted to the ICU. Median IVF volume administered in the first 3 hours was 2.0 L (IQR 2.0 L). Baseline characteristics and echocardiographic variables, stratified by DS2 and in-hospital death are listed in Table 1. BNP (only measured in 7 patients), mechanical ventilation, vasopressor use, and creatinine (the latter 3 included in SOFA score) were greater in the DS2 and non-survivor group.

**Table 1:** Characteristics of study participants.

	ΔSOFA ≥ 2 at 24 hours		In-Hospital Mortality	
	Yes (N = 17)	No (N = 56)	Yes (N = 17)	No (N = 56)
Age*	60.3 (12.3)	58 (15.46)	66.3 (12.9)	60.2 (14.7)
**Male**	8 (47, 26–69)	24 (43, 29–56)	4 (23, 1–46)	24 (43, 31–57)
**ED Systolic BP**	106 (36)	99.5 (37)	102 (33)	103.5 (39)
**ED Diastolic BP**	74 (26)	63.5 (26)	66 (24)	68.0 (27)
ED Heart Rate*	104 (23)	102 (24)	104 (21)	103 (24)
**ED Respiratory Rate**	22 (8)	18 (5)	20 (4)	20 (6)
**ED Sp0**_**2**_	96.5 (4.5)	98 (4)	98 (3)	98 (4)
**ED Temperature**	37.3 (1.3)	36.9 (1.5)	36.9 (2)	36.9 (1.2)
Weight (kg)*	75.2 (14.4)	73.3 (16.9)	70.6 (12.3)	74.5 (17.1)
**Past Medical History**				
**Diabetes**	9 (53, 31–73)	22 (39, 28–52)	10 (59, 36–78)	21 (38, 26–51)
**Stroke**	4 (24, 10–47)	12 (21, 12–34)	5 (29, 13–53)	11 (20, 11–32)
**End-Stage Renal Disease**	5 (29, 13–53)	14 (25, 15–38)	6 (35, 17–59)	13 (23, 14–36)
**Coronary Artery Disease**	6 (35, 17–59)	12 (21, 12–34)	4 (24, 10–47)	14 (25, 15–38)
**Cancer**	5 (29, 13–53)	21 (38, 26–51)	9 (53, 31–73)	17 (30, 19–43)
**COPD/Asthma**	5 (29, 13–53)	11 (20, 11–32)	4 (24, 10–47))	12 (21, 12–34)
**Heart Failure (pEF or rEF)**	3 (18, 6–41)	11 (20, 11–32)	5 (29, 13–53)	9 (16, 9–28)
**Echocardiographic Parameters**				
**LVEF (%): Hour 0**	55 (35)	55 (15)	45.0 (35)	55 (15)
**LVEF (%): Hour 3**	50 (30)	55 (20)	47.5 (40)	55 (15)
**ΔEF 0–3 hours**	0 (15)	0 (10)	0 (7.5)	0 (10)
**TAPSE (cm): Hour 0**	2.0 (1.2)	2.0 (0.75)	1.8 (0.5)	2.0 (0.9)
**TAPSE (cm): Hour 3**	2.1 (0.3)	2.0 (0.9)	2.2 (0.7)	2.0 (0.7)
**ΔTAPSE (cm): 0–3 Hours**	-0.1 (0.7)	-0.1 (0.5)	0.0 (0.2)	-0.2 (0.5)
**Avg e’ (m/s): hour 0**	0.08 (0.04)	0.07 (0.03)	0.06 (0.02)	0.08 (0.04)
**Avg e’ (m/s): hour 3**	0.06 (0.03)	0.08 (0.04)	0.06 (0.04)	0.08 (0.04)
**ΔAvg e’ (m/s) 0–3 hours**	0.01 (0.01)	0.0 (0.02)	0.01 (0.02)	0.0 (0.02)
**Laboratory Results & Interventions**				
**ED Lactate (mmol/L)**	3 (3.5)	3.15 (1.95)	4.3 (2.95)	2.9 (1.9)
**ED Troponin-I (ng/mL)**	0.08 (0.25)	0.04 (0.05)	0.07 (0.07)	0.05 (0.05)
**NT-pro BNP (pg/mL)**	1095 (932)	135.5 (107)	1562 (0)	180 (555)
**Hemoglobin (mg/dL)**	10.65 (3.75)	11.1 (4)	10.4 (2.55)	11.2 (4.1)
**Creatinine (mg/dL)**	3.26 (2.4)	1.91 (2.92)	2.69 (2.77)	2.05 (3.04)
**IVF 3 hours (L)**	2.0 (1.1)	2.0 (2.0)	2.0 (1.5)	2.0 (2.0)
**IVF 24 hours (ml)**	3650 (2000)	3000 (3000)	3600 (2800)	3145 (3100)
**30 cc/kg in 1**^**st**^ **3 Hours**	7 (41, 12–65)	31 (55, 42–71)	10 (59, 30–90)	28 (58, 37–65)
**SOFA score: ED**	6 (4)	3 (4)	5 (4)	3 (5)
**SOFA score: 24 hours**	10 (6.5)	2 (4)	6.5 (7)	2 (5)
**Mechanical Ventilation**	12 (71, 47–87)	7 (13, 6–24)	9 (53, 31–74)	10 (18, 10–30)
**Vasopressor use**	12 (71, 47–87)	7 (13, 6–24)	9 (53, 31–74)	10 (18, 10–30)
**Days in Hospital**	7 (11)	7 (7)	6 (10)	7 (7)
**Source of Infection** (%, N)				
**Pneumonia**	29 (5)	36 (20)	41 (7)	34 (19)
**GI/GU**	41 (7)	45 (25)	35 (6)	38 (21)
**Skin/Soft Tissue/Bone**	12 (2)	5 (3)	6 (1)	4 (2)
**Other**	18 (3)	14 (8)	18 (3)	25 (14)

Proportions are given as N (%, 95% CI); continuous variables given as median (inter-quartile range) except as denoted by *, which are given as mean (standard deviation). BP = blood pressure; COPD = chronic obstructive pulmonary disease; LVEF = left-ventricular ejection fraction; pEF/rEF = preserved/reduced LVEF; TAPASE = tricuspid annular plan systolic excursion; Avg e’ = average of septal & lateral mitral annular velocities; IVF = intravenous fluid; SOFA = sequential organ failure assessment; ED = emergency department; GI = gastrointestinal; GU = genitourinary; Other = indwelling vascular access device, bacteremia, or source unknown.

From 146 total echocardiograms, 13 were missing at least one e’ value (n = 8 in DS2 group), 12 were missing TAPSE (n = 7 in DS2 group), and 0 were missing LVEF. Missing ED SOFA values included Pa0_2_ in 44 patients (n = 7 in DS2 group) and bilirubin in 22 patients (n = 7 in DS2 group); at 24-hours: Pa0_2_ in 48 patients (n = 7 in DS2 group), and bilirubin in 51 patients (n = 8 in DS2 group).

**Fig 1:**
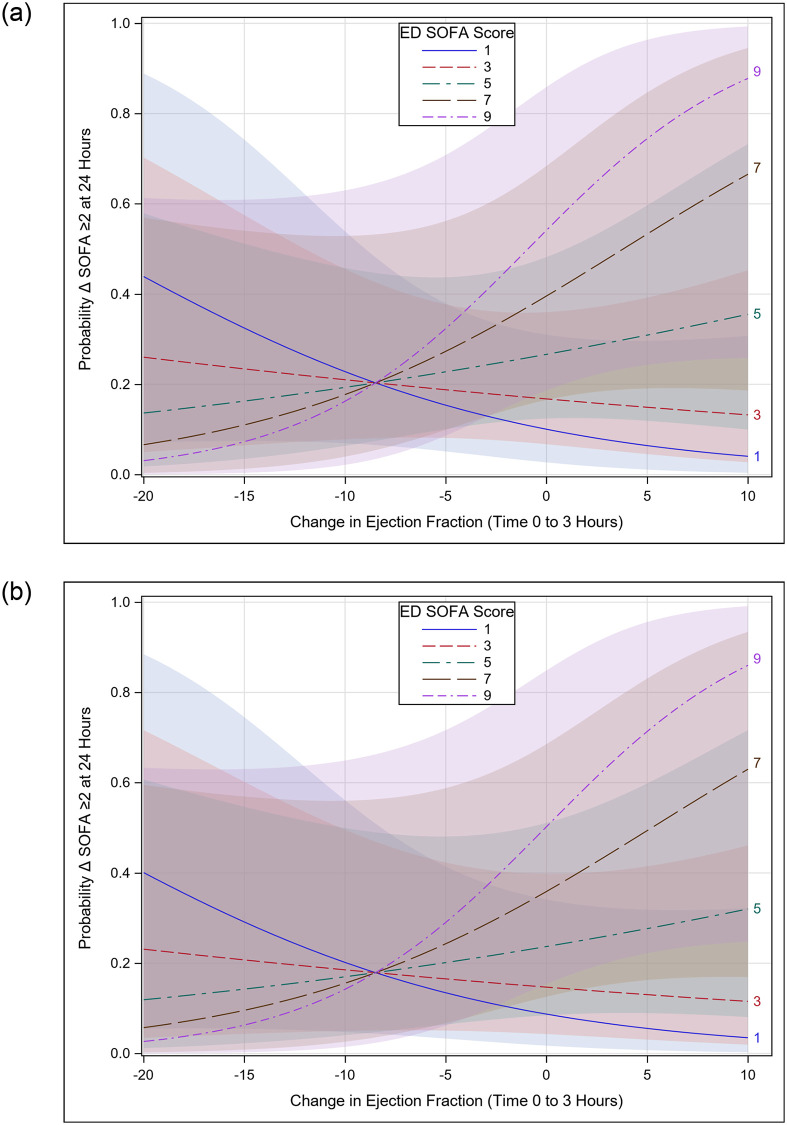
Interaction plot of change in LVEF from 0–3 hours with ED SOFA score. Interaction plot for showing that the influence of change in left ventricular ejection fraction on the probability of meeting the outcome of ΔSOFA ≥ 2 at 24 hours varies by baseline (ED) SOFA Score, adjusted for baseline inferior vena cava (IVC) collapsibility; bands represent 95% CIs. Panel A represents patients with IVC collapse ≥ 50%; Panel B represents patients with IVC collapse <50%.

For the primary outcome, e’ velocity was not associated with DS2, after adjustment for baseline (ED) SOFA score. Similarly, neither LVEF nor TAPSE were associated with DS2 (Table 2). Additionally, there was no adjusted association between Δe’ (0–3 hours) and DS2. Change in LVEF from 0–3 hours (ΔLVEF), adjusted for LVEF-ED SOFA score interaction and IVC collapse was associated with DS2, however. The relationship between ΔLVEF at 3 hours and DS2 is depicted graphically in Fig 1, stratified by IVC collapsibility, showing that the impact of ΔLVEF on the probability of DS2 varies by ED SOFA score, with minimal difference between IVC groups. For patients with greater ED SOFA scores, declining LVEF is associated with a lower probability of DS2 than static or increased LVEF, while the inverse relationship is seen with lower ED SOFA scores. A similar relationship was seen for ΔTAPSE (See S1 Fig in S1 File).

**Table 2:** Parameter estimates from logistic regression models.

	ΔSOFA ≥2 at 24 hours			In-Hospital Mortality		
	OR (CI)	SE	AUC (CI)	OR (CI)	SE	AUC (CI)
**PREDICTORS**						
E’ velocity	0.04 (0.001–1.11)	1.87	0.81 (065.-0.97)	1.15 (0.04–37.7)	1.77	0.86 (0.75–0.97)
IVC Collapse	0.63 (0.12–3.43)	0.43		0.09 (0.01–0.60)	0.49	
ED SOFA	1.62 (1.13–2.34)	0.19		1.44 (0.97–2.14)	0.20	
LVEF*	0.91 (0.74–1.11)	0.02	0.65 (0.45–0.85)	0.76 (0.60–0.96)	0.02	0.76 (0.59–0.94)
IVC Collapse	1.01 (0.25–4.10)	0.35		0.35 (0.07–1.71)	0.40	
ED SOFA	1.21 (0.94–1.54)	0.13		1.2 (0.89–1.50)	0.14	
TAPSE^	1.96 (0.36–10.73)	0.86	0.66 (0.44–0.88)	1.34(0.64–2.9)	1.26	0.81 (0.42–1.0)
IVC Collapse	1.34 (0.12–15.69)	0.63		0.23 (0.01–7.74)	0.89	
ED SOFA	1.24 (0.85–1.82)	0.19		2.0 (1.01–3.94)	0.35	

Each row represents a unique, 3 variable logistic regression model. SOFA = Sequential Organ Failure Assessment Score; LVEF = left ventricular ejection fraction; TAPSE = tricuspid annular plane systolic excursion; OR = odds-ratio; CI = 95% confidence interval; AUC = area under receiver-operating characteristic curve.

*OR is per 5% increase in LVEF.

^OR is per 5mm increase in TAPSE.

**Fig 2:**
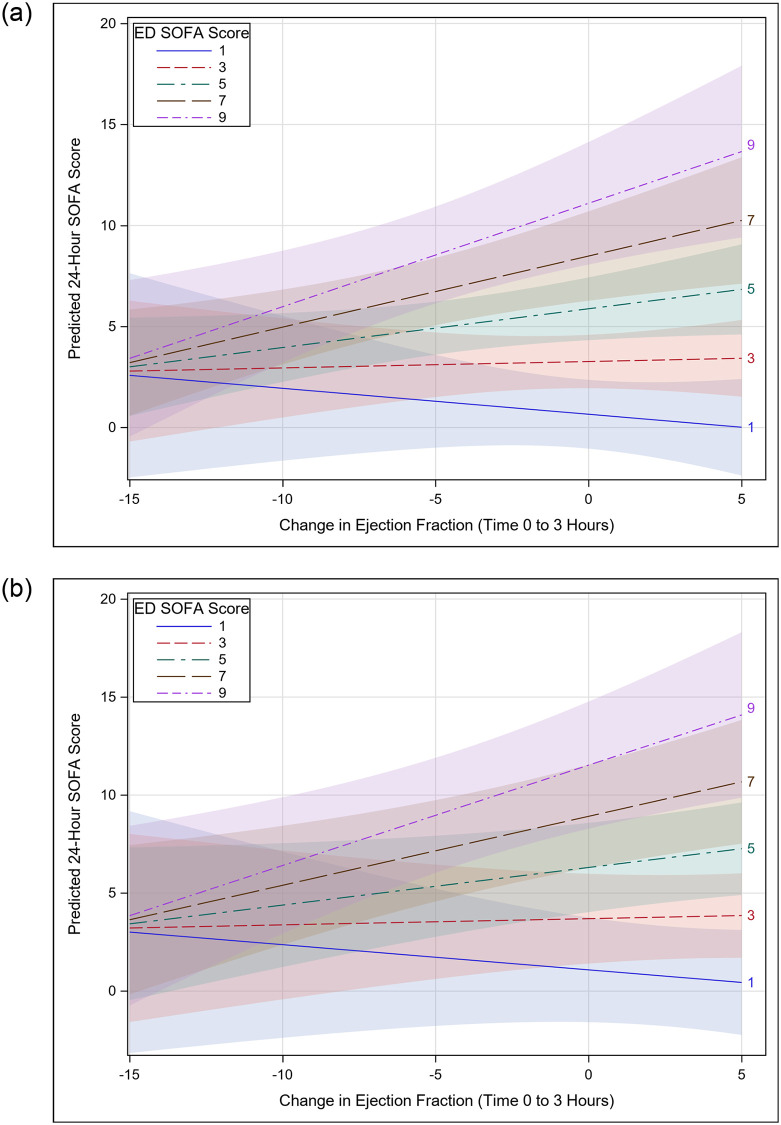
Interaction plot of change in LVEF from 0–3 hours with ED SOFA score. Interaction plot showing that the influence of change in left-ventricular ejection fraction on predicted SOFA Score at 24-hours varies by baseline (ED) SOFA Score, adjusted for age, troponin-I, Average change in E/e’ from 0-3hrs, and baseline inferior vena cava (IVC) collapsibility; bands represent 95% CIs. Panel A represents patients with IVC collapse ≥ 50%; Panel B represents patients with IVC collapse <50%.

For the outcome of 24-hour SOFA score, the final multivariable model included ΔLVEF at 3 hours, ED SOFA score, ED troponin-I value, age, average ΔE/e’ at 3 hours, IVC collapsibility, and interaction between ΔLVEF and ED SOFA score (Table 3). The impact of ΔLVEF on 24-hour SOFA score varied by ED SOFA score, as shown in Fig 2: when ED SOFA score is ≥ 5, greater decline in LVEF is associated with lower 24-hour SOFA score compared to LVEF that remains static or increases. For lower ED SOFA scores, the impact of ΔLVEF on 24-hour SOFA score declines until the relationship inverts (at ED SOFA ~3) and greater declines in LVEF are associated with greater 24-hour SOFA score and increased LVEF is associated with lower 24-hour SOFA score. Minimal difference was seen between IVC groups. The relationship between ΔTAPSE at 3-hours and 24-hour SOFA score was similar (see S1 Table, S2 Fig in S1 File).

**Table 3:** Linear model for 24-hour sofa prediction.

	SOFA Score at 24 hours			
**ΔLVEF Model** (p = 0.001)*		Estimate	SE	p-value
	**Predictor**			
	**ΔLVEF 0–3 hours**	-0.21	0.17	0.25
	**ED SOFA Score**	1.31	0.22	<0.001
	ΔLVEF*ED SOFA	0.08	0.03	0.02
	**ΔE/e’0–3 hours**	-0.25	0.14	0.09
	**Age** (per year)	0.11	0.04	0.03
	**IVC Collapse ≥50%**	-0.43	1.25	0.74
	**Troponin-I** ^$^	-0.36	0.91	0.69

*p-value for overall model.

^$^parameter estimate is per 1 ng/ml increase.

SOFA = sequential organ failure assessment; LVEF = left ventricular ejection fraction; ED = emergency department; IVC = inferior vena cava.

For the exploratory analysis, only LVEF on 0HrBE showed a significant crude association with in-hospital mortality (odds ratio (OR) 0.75 (CI 0.61–0.93) per 5% increase in LVEF. Adjusting for ED SOFA score and baseline IVC collapse yielded an OR of 0.76 (CI 0.60–0.96) per 5% increase in LVEF, AUC 0.76 (CI 0.59–0.94).

Median volume of IVF administered in the first 3 hours of treatment, and the proportion who received 30cc/kg, was similar amongst patients whose LVEF and TAPSE increased versus decreased during this time (S2 Table in S1 File).

## Discussion

In this prospective observational study, we found no association between diastolic function at ED arrival, or change from 0–3 hours, and probability of ≥2-point increase in SOFA score in patients with sepsis. However, changes in LVEF and TAPSE during the initial ED resuscitation were associated with illness severity at 24 hours. For patients with lower ED SOFA score, early improvement in RV and LV function is associated with better outcomes at 24 hours. The opposite relationship was observed in patients with more severe illness at presentation, who were found to have substantially worse outcomes at 24 hours when LV and RV function improve. We also found that reduced LVEF at presentation is independently associated with in-hospital mortality. To our knowledge, this is the first prospective description of the association between echocardiographic measures and outcomes in unresuscitated septic patients [3]. While these results are novel, they should be viewed as preliminary, hypothesis-generating findings that require external validation.

We found no association between e’ velocity and increased 24-hour sepsis severity. We hypothesized that the deleterious effect of cardiac dysfunction on outcomes was mediated through impaired relaxation, of which e’ is a measure. Intrinsic reduction in relaxation would be exacerbated by IVF administration via increased LV filling pressure, potentially accounting for some of the harms of volume overload. That no such association was found may reflect that fact that our study was underpowered and/or had too few events to detect a signal, or that the timeframe of reassessment (3 hours) was too short to identify significant changes. Adequately powered studies are needed to confirm these findings.

We did find that patients with greater ED SOFA scores had reduced probability of DS2 when LV and RV systolic function declined from 0–3 hours, compared to static or increased function. The etiology of this observed relationship remains unknown. One potential explanation is a component of SC that includes hibernating myocardium. Hibernating myocardium, in which myocardial dormancy during extreme physiologic stress, is theorized to help cells resume normal function and survive until the stressor (i.e., sepsis) ends. This phenomenon occurs in ischemic myocardium and in heart failure as a response to metabolic and hemodynamic stress [26, 27]; its presence in sepsis is suggested by paucity of myocardial cell death in patients who have died from infections [28, 29]. More severe presentations, as indicated by increased SOFA score, would arguably be expected to induce more hibernating myocardium (manifested as declining systolic function), as we observed. However, this is but one of many plausible explanations and data from our small, observational study cannot be used to draw firm conclusions.

Our results also suggest that additional factors may influence cardiac function. In patients with low baseline SOFA score—indicating less metabolic/hemodynamic stress—the impetus to incite hibernating myocardium may be reduced. Thus, changes in cardiac function could occur due to different mechanisms influenced by patient- and disease-specific factors. Further study of SC is needed to confirm these findings and clarify the mechanisms.

The same relationships between change in LV and RV systolic function from 0–3 hours were seen for the outcome of 24-hour SOFA score, adjusted for age, Troponin, heart failure history, and average ΔE/e’ from 0–3 hours. This appears to support our finding that ED assessment of cardiac function may provide independently useful prognostic data. BNP was greater in non-survivors, consistent with prior literature [3], but it was measured in a small number of patients thereby leading to worsened model fit, so it was excluded. We also found that ED lactate did not provide additive value to SOFA score, consistent with prior studies [30].

Our data suggests an association between dynamic changes in cardiac function during the first 3 hours of resuscitation with increased illness severity at 24 hours, independent of IVF administration. Overall IVF volumes were modest, and minimal differences in absolute volumes were noted in various subgroups. Proportions receiving at least 30cc/kg of IVF were similar in patients with declining versus non-declining LVEF (46 versus 51%). The impact of large volume IVF administration is important given that 30cc/kg is currently recommended in patients with hypoperfusion, but fluid overload is associated with adverse outcomes [31–34].

At presentation, only LVEF was significantly associated with in-hospital mortality. The utility of this finding by itself is uncertain, as reduced LVEF may simply be an indicator of poor baseline health. When adjusted for SOFA score, the standard for severity-of-illness stratification in sepsis [35], and IVC collapsibility, the relationship remained statistically significant (OR of death, 0.76 (CI 0.60–0.96) per 5% increase in LVEF). Clinical heterogeneity in the sepsis population, and overall outcomes, are widely recognized, but early identification of those who will do poorly is challenging and we therefore feel that further exploration of the prognostic value of LVEF is warranted.

Our data represent novel but extremely preliminary work and as such our results require external confirmation. Studies attempting to do so should concurrently seek to identify biomolecular mediators of myocardial depression. Further elucidation of the natural history of SC and its clinical correlates are necessary to improve prognostication and develop targeted interventions. If our results can be confirmed, future studies should expand on our findings to assess cardiac functional change throughout the remaining resuscitative and recovery phases of the disease, how treatments impact these changes, and whether some of the deleterious effects of volume overload are mediated by the complex interplay of central venous hypertension, cardiac dysfunction, and IVF administration.

## Limitations

Several limitations are worth noting for this study. This was an exploratory, observational study conducted on a convenience sample of patients. Thus, there was patient-level heterogeneity in treatments administered and no objective measures of whether said treatments were appropriate or adequate. Sample size was small, we were underpowered for our primary outcome, and the low number of in-hospital deaths and DS2 events limited the number of confounders we could include in multivariable adjustment. As such, our results must be viewed as preliminary as adequately-powered studies may detect associations that we could not. Pre-septic cardiac function was unknown, precluding elucidations of the impact of sepsis itself on myocardial performance. Assessment of relative change from 0–3 hours mitigates this to some extent, but does not account for the fact that patients are likely to present at different phases of their illness. While these latter issues may represent intractable problems in diagnostic studies of SC, they do not preclude the use of echocardiography data at presentation in prognostic studies [36]. While we adjusted for volume status/preload in our statistical models, this may not adequately address loading conditions on the heart, which can alter echocardiographic measures of cardiac function. That nearly 20% of patients in our cohort had a documented history of heart failure may also introduce confounding. Whether the observed changes in cardiac function and the association with outcomes would be similar for those with, versus without, such history is not an issue our study can answer given our limited sample size. One might consider patients with a history of heart failure to have worse baseline health and thus be expected to have overall worse outcomes. Of the parameters investigated, we found only measures of systolic function to be associated with increasing illness severity and mortality. However, the complexity of heart failure is belied by our simplified, binary definition (“yes” versus “no”) and thus delineation of the true relationship requires further study in larger studies with more complete description of type and degree of heart failure. Finally, all our patients were African American, reflecting the community which our hospital serves, and thus the results may not apply to other ethnic groups.

## Conclusions

In the first prospective study of unresuscitated ED patients with suspected sepsis, we found that change in LV and RV systolic function from 0–3 hours was associated with illness severity at 24-hours, with the direction and magnitude of change dependent on the initial severity, as measured by ED SOFA score. We also found that reduced LVEF was associated with increased odds of in-hospital death even after adjustment for severity of illness. While preliminary, these findings add important data to the pursuit of improved understanding of the natural history and clinical implications of SC.

## Supporting information

S1 File Supplementary methods, results, tables and figures. (DOCX)

S1 Checklist STROBE statement—Checklist of items that should be included in reports of *cohort studies*. (DOCX)
