# Supplementary material for: Early echocardiographic assessment of cardiac function may be prognostically informative in unresuscitated patients with sepsis: A prospective observational study
Source: PLoS One. 2022 Jul 8;17(7):e0269814. doi: 10.1371/journal.pone.0269814 (PMC9270056; doi:10.1371/journal.pone.0269814)
Supplement: S1 File — (DOCX) [file pone.0269814.s001.docx]

**SUPPLEMENTARY METHODS**

Cardiac Image Acquisition

Patients were scanned recumbent or in the left lateral decubitus position if needed to optimize image acquisition. Images of the heart were obtained in the parasternal long-axis view, parasternal short-axis view (at the level of the mitral valve and papillary muscles) and the apical four-chamber (A4C), two-chamber (A2C), long-axis (A3C), and A4C right-ventricular (RV) focused views. From the A4C view, trans-mitral inflow velocity was obtained using pulsed-wave Doppler with the sample volume placed at the tips of the mitral valve leaflets. Tissue Doppler imaging was used to measure velocity of the septal and lateral mitral annulus in accordance with guidelines from the American Society for Echocardiography (ASE) [19]. The inferior vena cava (IVC) was imaged in the long axis from the subcostal approach. For each view, a minimum of 2 three-beat clips was stored. Each hemothorax was imaged to a depth of 16cm in the anterior-superior zone to assess for the presence of B-lines. Images were stored locally on the ultrasound system and transmitted to an EchoPAC Workstation (GE Healthcare, Milwaukee, WI) for offline analysis. Treating clinicians could perform their own ultrasound examinations but they were blinded to study echocardiogram results with the exception of critical findings (e.g., large pericardial effusion) potentially requiring urgent intervention.

Echocardiography Interpretation

Left ventricular ejection fraction (LVEF) was determined by visual estimation in increments of 5% after review of parasternal and apical images. For LV diastolic function, peak early (E) and late (A) trans-mitral inflow velocity was measured from the pulsed-wave spectral tracing, except in cases where E-A fusion precluded identification of individual waveforms or the mitral valve was abnormal (e.g., stenotic or heavily calcified). Peak velocity of the mitral annulus was measured at both the septal (e’_s_) and lateral (e’_l_) positions; when isolated ectopic beats were present, 3 consecutive beats were measured and the average velocity was taken, and a 6-beat average was used for patients in atrial fibrillation. The E/e’ ratio (a surrogate of estimated LV filling pressure) was calculated for septal, lateral, and average mitral annular velocities. Right ventricular systolic function was assessed using tricuspid annular plane systolic excursion (TAPSE) obtained with the “Auto M-mode” feature on the EchoPAC system with the cursor placed at the lateral tricuspid annulus. IVC diameter was measured at inspiration (minimum diameter) and expiration (maximum diameter).

Statistical Analysis

For logistic regression models, an events-per-variable ratio of ~10:1 was used; model fit and discrimination were assessed using McFadden’s R^2^ and area under the receiver operating characteristic curve (AUC).

For each GLM, the normality assumption of residual distribution was assessed by visual inspection of QQ plots and multicollinearity between continuous variable was assessed using variance inflation factor; model fit was assessed using adjusted R^2^ and Akaike’s information criteria (AIC).

Results

**TAPSE ASSESSMENTS**

Change in TAPSE from 0-3hrs, adjusted for ED SOFA score and IVC collapsibility, was associated with DS2, AUC 0.75 (CI 0.38-1.0). The relationship depicted graphically in supplemental Figure 1. showing that the probability of DS2 varies by ED SOFA score.

A multivariable GLM was constructed, with ∆TAPSE substituted for ∆LVEF, and the following covariates: ED SOFA score, ED troponin-I value, age, change in avererage E/e’, IVC collapsibility, and interaction between ∆TAPSE and ED SOFA score (p=0.06 for the interaction). As shown in Supplemental Figure 2, patients with greater ED SOFA scores (> ~5) have lower 24-hours SOFA scores when TAPSE declines compared to when it remains static or increases. For lesser ED SOFA scores, the relationship is inverted—declining TAPSE is associated with greater 24-hour SOFA score compared whereas static or increase is associated with lesser scores at 24-hours.

Supplemental Table 1

| Supplemental Table 1: Linear Model for 24-Hour SOFA Prediction | | | | |
| --- | --- | --- | --- | --- |
|  | **SOFA Score at 24 hours** | | | |
| ∆TAPSE Model p=0.06)* |  | Estimate | SE | p-value |
|  | **Predictor** |  |  |  |
|  | **∆TAPSE 0-3 hours** | -10.29 | 4.46 | 0.07 |
|  | **ED SOFA Score** | 1.34 | 0.21 | 0.01 |
|  | **∆TAPSE*ED SOFA** | 1.79 | 0.62 | 0.06 |
|  | **IVC Collapsibility ≥50%** | -1.12 | 1.42 | 0.49 |
|  | **Age** (per year) | 0.10 | 0.07 | 0.27 |
|  | **∆E/e’0-3 hours** | 0.06 | 0.19 | 0.78 |
|  | **Troponin-I^$^** | -2.12 | 1.35 | 0.22 |

*p-value for overall model

$parameter estimate is per 1 ng/ml increase

Table Legend: SOFA= sequential organ failure assessment; ED= emergency department; TAPSE= tricuspid annular plane systolic excursion; IVC=inferior vena cava.

Supplemental Table 2

| Supplemental Table 2: Fluid Administration by Change in Left & Right Ventricular Function | | | | |
| --- | --- | --- | --- | --- |
|  | **∆EF at 3 hours < 0 (n=28)** | **∆EF at 3 hours ≥ 0 (n=45)** | **∆TAPSE at 3 hours < 0 (n=41)** | **∆TAPSE at 3 hours ≥ 0 (n=32)** |
| IVF Volume (L) 1^st^ 3 Hours | 2.0 (2.4) | 1.9 (2.0) | 2.0 (1.4) | 1.0 (2.5) |
| 30cc/kg IVF 1^st^ 3 Hours* | 13 (46) | 23 (51) | 16 (39) | 9 (28) |
| ∆Average E/e’ at 3 Hours | 1.41 (2.41) | -1.47 (3.96) | 0.02 (1.9) | -1.4 (6.2) |

Figure Legend: continuous variables given as median (interquartile range); *proportions as N (%). IVF= intravenous fluids; EF= left ventricular ejection fraction; TAPSE= tricuspid annular plane systolic excursion.

Supplemental Figure 1

**A.**


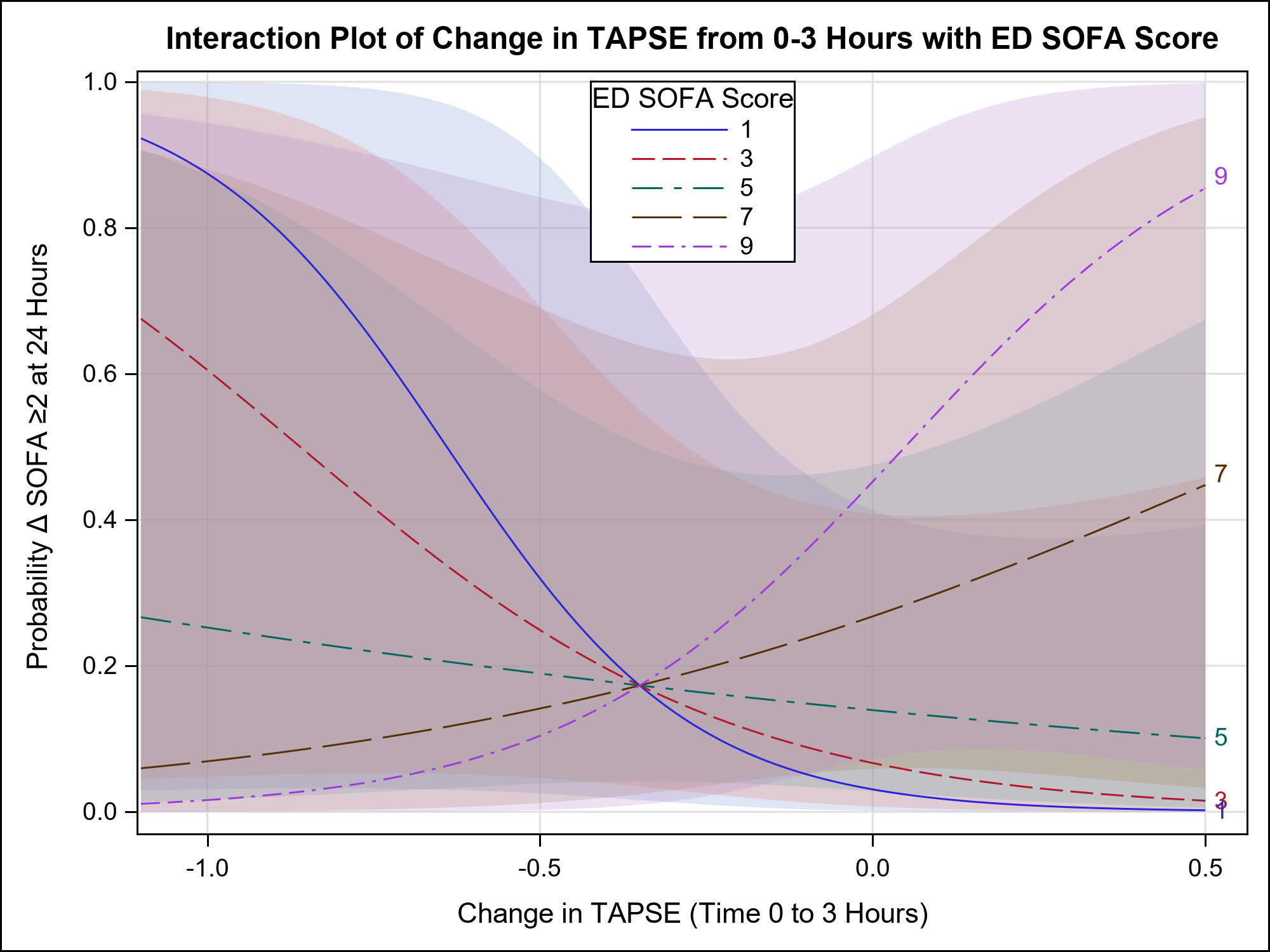


**B.**


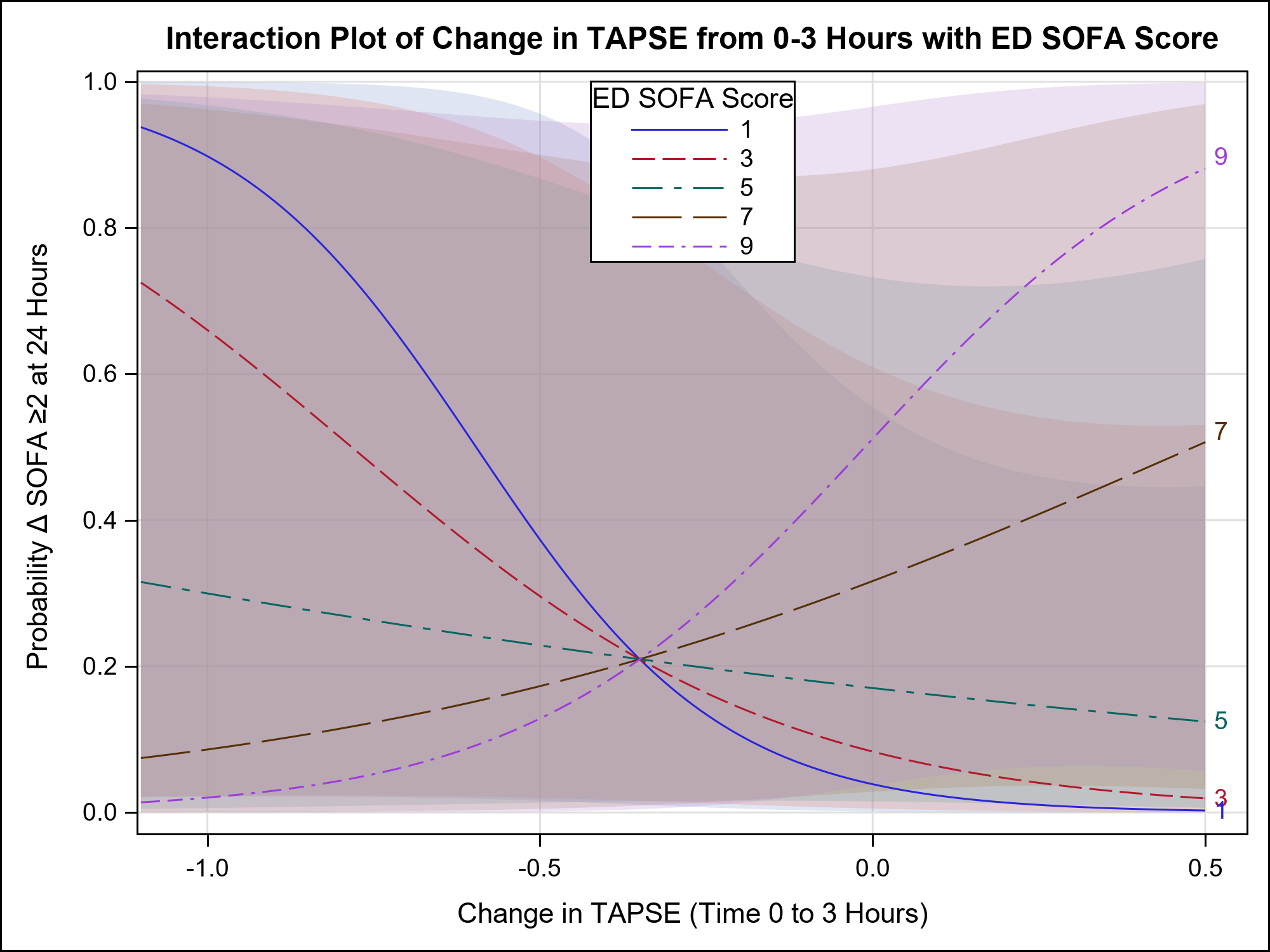


Legend: Interaction plot for showing that the influence of change in tricuspid annular plane systolic excursion (in centimeters) on the probability of meeting the outcome of ∆SOFA ≥ 2 at 24 hours varies by baseline (ED) SOFA Score, adjusted for baseline IVC collapsibility; bands represent 95% CIs. Panel A represents patients with IVC collapse ≥ 50%; Panel B represents patients with IVC collapse <50%.

Supplemental Figure 2

**A.**


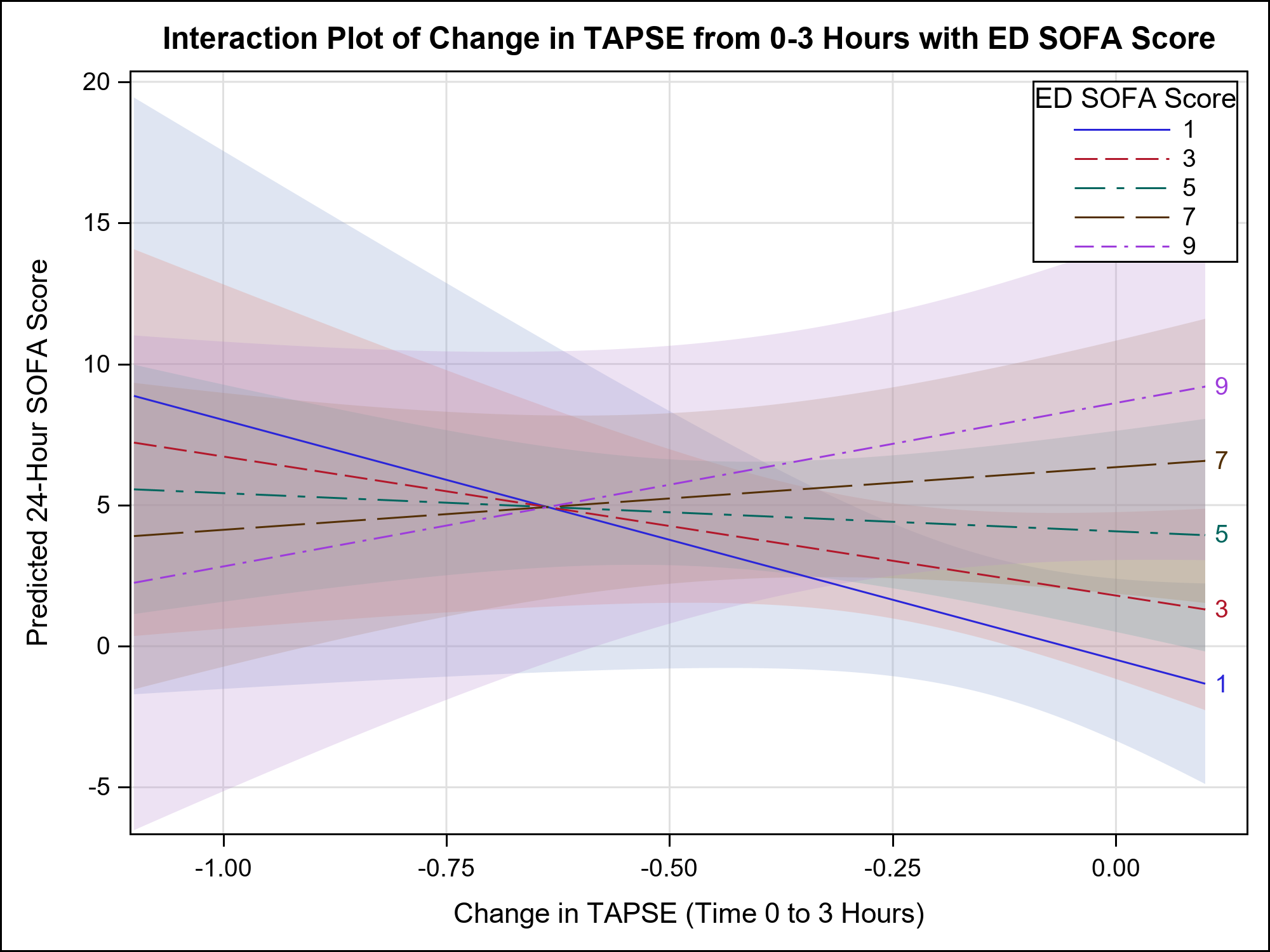

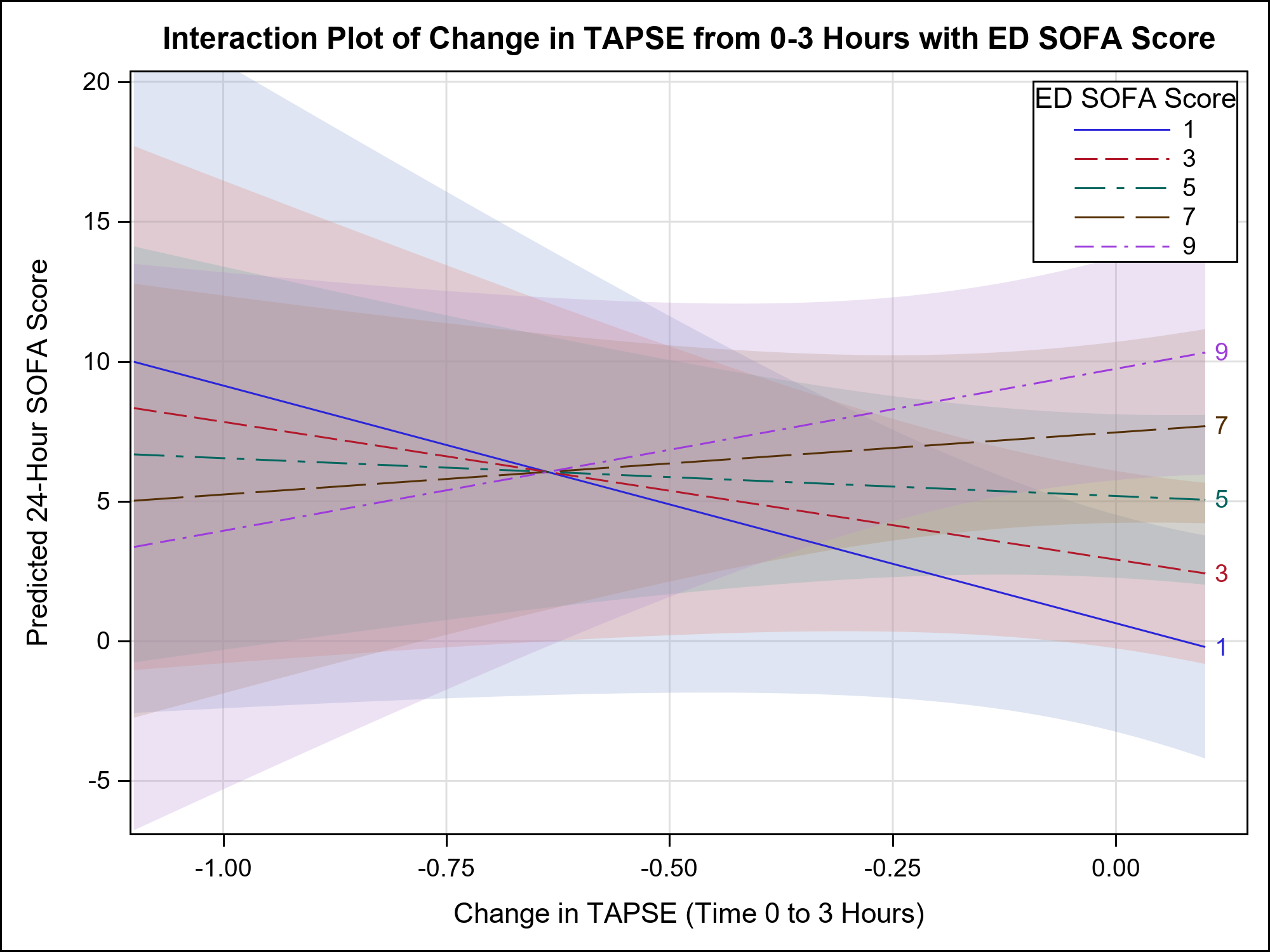


**B.**

Legend: Interaction plot showing that the influence of change change in tricuspid annular plane systolic excursion (in centimeters) on predicted SOFA Score at 24-hours varies by baseline (ED) SOFA Score, adjusted for age, troponin-I, Average change in E/e’ from 0-3hrs, and baseline inferior vena cava (IVC) collapsibility; bands represent 95% CIs. Panel A represents patients with IVC collapse ≥ 50%; Panel B represents patients with IVC collapse <50%.
